# c-Myc transactivates GP73 and promotes metastasis of hepatocellular carcinoma cells through GP73-mediated MMP-7 trafficking in a mildly hypoxic microenvironment

**DOI:** 10.1038/s41389-019-0166-7

**Published:** 2019-10-07

**Authors:** Yiming Liu, Sining Zhou, Jieyao Shi, Xiaodi Zhang, Linhui Shentu, Zhi Chen, Linfu Zhou

**Affiliations:** 1 State Key Laboratory for Diagnosis and Treatment of Infectious Diseases, Collaborative Innovation Center for Diagnosis and Treatment of Infectious Disease, The First Affiliated Hospital, Zhejiang University School of Medicine, Hangzhou, 310003 China; 2 Department of Biochemistry and Molecular Biology, Zhejiang University School of Medicine, Hangzhou, 310058 China; 3 Key Laboratory of Clinical In Vitro Diagnostic Techniques of Zhejiang Province, Department of the First Affiliated Hospital, School of Medicine, Zhejiang University, Hangzhou, 310003 China; 4 College of Pharmaceutical Sciences, Zhejiang University, Hangzhou, 310058 China

**Keywords:** Cancer microenvironment, Cell migration

## Abstract

Golgi phosphoprotein 73 (GP73), encoded by *GOLM1*, is a highly expressed factor in hepatocellular carcinoma (HCC) cells and has been regarded for several years as a remarkable serum biomarker for the diagnosis of HCC. Recently, it was found that upregulation of GP73 promotes cancer metastasis, but the mechanism is complex, and it is even unclear how the gene is transactivated in HCC cells. In this study, it was discovered that c-Myc transactivated GP73 in a mildly hypoxic microenvironment and that the activation of c-Myc upregulated the expression of matrix metalloproteinase-7 (MMP-7). Moreover, it is shown that GP73 interacted with intracellular MMP-7 in the region of the cytoplasmic domain and facilitated the trafficking and secretion of MMP-7, resulting in cell metastasis. This study indicates that GP73 is transactivated by c-Myc and serves as a transporter in the trafficking of intracellular MMP-7 in HCC cells. These findings suggest that GP73 is a potential target for combating metastatic HCC.

## Introduction

Hepatocellular carcinoma (HCC) is the fifth most common carcinoma and the third leading cause of cancer-related death in the world^1^. It is known that >80% of HCCs are caused by hepatitis B virus (HBV) or hepatitis C virus (HCV), especially in China and other Asian countries^2,3^. Since the high recurrence and metastasis rate of HCC, metastasis is the main cause of death related to this type of cancer. Metastasis is the main cause of HCC-related death because hypoxia and plentiful tumor vessels create a tumor microenvironment that facilitates the recurrence and metastasis of HCC^4–6^.

*MYC* is a critical gene controlling cell proliferation and development, and c-Myc, one of its transcripts, plays critical roles in most cancers^7,8^. Abnormal expression of c-Myc promotes tumor proliferation and facilitates the process of hypoxia resistance^9^. In recent studies, it has been reported that abnormal expression of c-Myc also promotes cancer metastasis, but the mechanism is complex and remains to be elucidated^10–12^.

Golgi phosphoprotein 73 (GP73) is a transmembrane protein encoded by *GOLM1* and is located in the *cis*-Golgi cisternae^13,14^. As previously reported, GP73 is highly expressed in most types of cancers, especially in HCC, and it has been adopted clinically as a cancer biomarker for decades^15,16^. In most recent studies, it was discovered that high expression of GP73 facilitates cancer metastasis by promoting epithelial-mesenchymal transition (EMT), and evidence has implicated that GP73 is not only a serum biomarker but also an important player in carcinogenesis and metastasis^17–19^. Viral infection is reportedly to activate the expression of GP73 in HCC cells; however, it has also been indicated that GP73 is upregulated in cancer cells without viral infection, such as non-viral-related HCC, bladder cancer and prostate cancer^20–22^. Nevertheless, how GP73 is upregulated in cancer cells without viral infection remains unidentified.

In this study, we discovered that upregulation of c-Myc promoted transactivation of GP73 in a mildly hypoxic tumor microenvironment and GP73-mediated trafficking of intracellular MMP-7, resulting in HCC metastasis. These findings demonstrate that GP73 is highly expressed in HCC cells and reveal the mechanism involved in tumor metastasis in a mildly hypoxic microenvironment. The study will be important for exploring new targets in combating HCC metastasis.

## Results

### Upregulation of c-Myc and GP73 promotes cell metastasis in a mildly hypoxic microenvironment

**Fig. 1:**
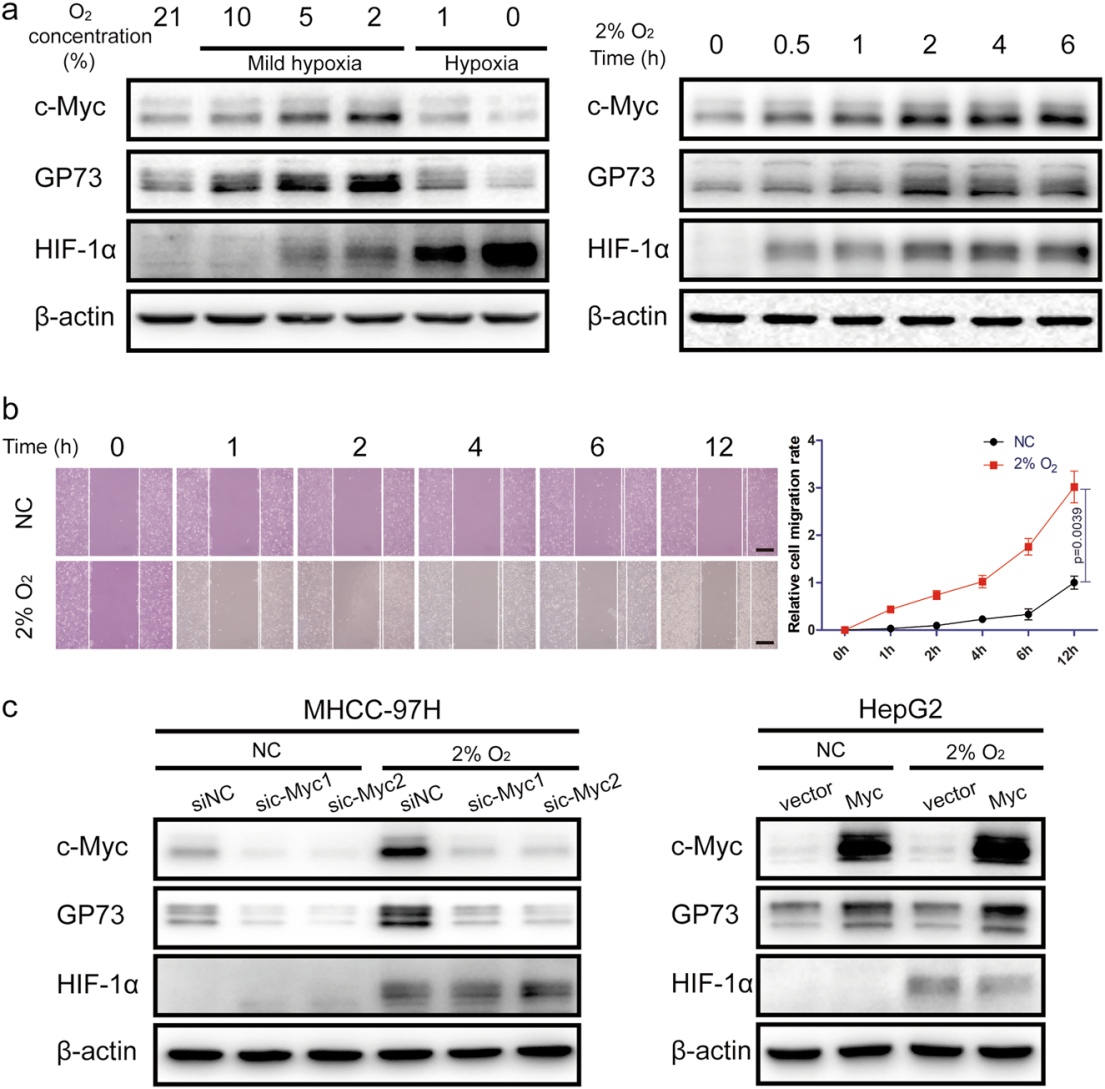
Upregulation of c-Myc and GP73 promotes cell metastasis in a mildly hypoxic microenvironment. **a** HepG2 cells were incubated in a hypoxic chamber containing 0%, 1%, 2%, 5%, 10% or 21% oxygen for 6 h before samples were harvested. Then, HepG2 cells were incubated in a hypoxic chamber containing 2% oxygen for 0, 0.5, 1, 2, 4 or 6 h before samples were harvested. The levels of the indicated proteins were determined by immunoblotting. **b** MHCC-97H cells were grown in monolayers and treated with serum-free DMEM for 12 h. Cells were wounded using 10 μL micropipette tips, and the cells in the hypoxia group were cultured in hypoxic chambers containing 2% oxygen. The cells in the control group were cultured in hypoxic chambers containing 21% oxygen. Images were captured 0, 1, 2, 4, 6 or 12 h after treatment (scale bar: 100 μm). **c** HepG2 cells were transfected with 2 μg pCMV-c-Myc or pCMV vector. MHCC-97H cells were transfected with c-Myc-specific siRNAs or control siRNAs. After 48 hh of transfection, the cells were treated with 2% or 21% oxygen for an additional 6 h before the samples were harvested. The levels of the indicated proteins were determined by immunoblotting. Data in **b** are the mean ± s.e.m. and represent three independent experiments. A two-tailed Student’s *t*-test was used for statistical analysis

In our previous studies, it was discovered that GP73 and c-Myc were upregulated synchronously in a mildly hypoxic microenvironment. In order to probe the relationships between c-Myc and GP73, HepG2 cells were cultured under hypoxic conditions with different oxygen concentrations for 6 h. The results of quantitative real-time PCR (qRT-PCR) and immunoblotting analysis demonstrated that the expression of c-Myc and GP73 was upregulated under mildly hypoxic conditions (oxygen concentration from 21 to 2%), and the highest peak of c-Myc expression emerged at an oxygen concentration of 2%. However, under hypoxic conditions (oxygen concentration from 1 to 0%), the expression of both c-Myc and GP73 were reduced. Cells were then cultured in a CO_2_ incubator containing 2% O_2_ for different treatment durations, and the results showed that both c-Myc and GP73 increased 6 h after stimulation (Fig. 1a, S1a). To investigate whether mildly hypoxic conditions facilitated cell migration, the wound healing assay was performed. The results revealed that cell migration was strongly enhanced under conditions of 2% O_2_, which implicated that mildly hypoxic circumstances promoted cancer metastasis (Fig. 1b). As mentioned before, c-Myc and GP73 were upregulated in HepG2 cells incubated in mildly hypoxic conditions of 2% O_2_, and it was reported that upregulation of both c-Myc and GP73 facilitated cancer metastasis; we posit that mildly hypoxic conditions promoted cell migration via upregulation of c-Myc and GP73. The expression of c-Myc was mediated using a c-Myc expression vector and an shRNA vector targeting c-Myc. The results of qRT-PCR, immunoblotting and wound healing assays showed that upregulation of c-Myc promoted cell invasion, especially under mildly hypoxic conditions, and, strikingly, c-Myc was correlated positively with GP73 (Fig. 1c, S1b-d), which suggested that c-Myc might have a modulatory effect on GP73 expression.

These findings indicated that the upregulation of c-Myc and GP73 facilitated cell migration in a mildly hypoxic microenvironment and that c-Myc might be involved in the regulation of GP73.

### c-Myc positively modulates GP73 expression and promotes tumor progression in vivo

**Fig. 2:**
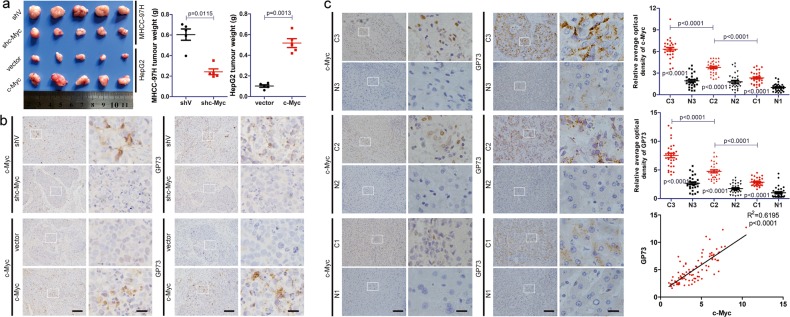
c-Myc positively modulates GP73 expression and promotes tumor progression in vivo. **a** Tumors derived from nude mice bearing xenografts of HepG2^vector^, HepG2^c-Myc^, MHCC-97H^shV^ and MHCC-97H^shc-Myc^ cells. The tumor weights of xenografts are indicated using scatter plots. **b** Immunohistochemical analysis of c-Myc and GP73 in tumors derived from nude mice bearing xenografts (scale bar: left 50 μm, right 10 μm). **c** Immunohistochemical analysis of c-Myc and GP73 in primary tumor (*n* = 90) and adjacent liver (*n* = 90) tissues of HCC patients. Images are labeled by stage (1, 2, 3) and tissue type (C = primary tumor, *N* = adjacent liver; scale bar: left 50 μm, right 10 μm). The expression of c-Myc and GP73 was measured using ImageJ and represented using average optical density (AOD). The scatter plot indicates the relative expression of c-Myc and GP73. The correlation between c-Myc and GP73 in primary tumor tissues was represented using linear correlation. Data in **a**, **b** and **c** are the mean ± s.e.m. A two-tailed Student’s *t*-test was used for statistical analysis

To further investigate the relationship between c-Myc and GP73 in vivo, we constructed HepG2 cells stably expressing c-Myc and MHCC-97H cells with stable knockdown of c-Myc. In consistent with previous reports, xenograft models and Ki-67 staining showed that overexpression of c-Myc promoted tumor proliferation and that knockdown of c-Myc inhibited tumor proliferation in vivo (Fig. 2a, S2a, b). Moreover, immunohistochemical analysis demonstrated that c-Myc positively regulated GP73 expression, which further supported that c-Myc regulated GP73 expression (Fig. 2b).

Therefore, it was hypothesized that c-Myc correlated positively with GP73 in HCC-related tissues. Immunochemical analysis of c-Myc and GP73 was performed on primary tumor tissues and adjacent liver tissues derived from HCC patients (*n* = 90). In comparison with adjacent liver tissues, both c-Myc and GP73 were overexpressed in primary tumor tissues. It was also discovered that the expression of c-Myc and GP73 increased as the disease progressed. Strikingly, c-Myc correlated positively with GP73 in both tumor tissues and adjacent liver tissues (*R*^2^ = 0.6195) (Fig. 2c).

The data above revealed that c-Myc positively modulated GP73 expression and promoted tumor progression in vivo and that c-Myc correlated positively with GP73 in HCC-related tissues, which confirmed that c-Myc regulated GP73 expression.

### Upregulation of c-Myc promotes transactivation of GP73

**Fig. 3:**
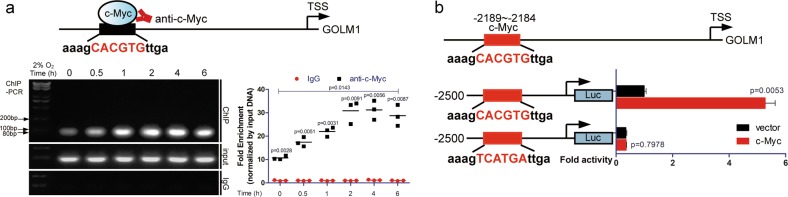
Upregulation of c-Myc promotes transactivation of GP73. **a** The products of ChIP-PCR in the input, IgG and ChIP groups were analyzed using agarose gel electrophoresis. The abundance of DNA fragments was measured using ImageJ and is represented using AOD. **b** Luciferase activity was measured after HepG2 cells were transfected with pGL4.19 containing promoter of *GOLM1*/binding site mutated mutant, pRL-TK and vector/c-Myc. Data in **a** and **b** are the mean ± s.e.m., and the data are representative of three independent experiments. A two-tailed Student’s *t*-test was used for statistical calculation

Considering the fact that c-Myc is an important transcription factor in tumor progression and c-Myc positively regulates GP73 expression, it could be hypothesized that c-Myc targets the promoter of *GOLM1* and activates its transcription. Based on the data of the JASPAR database, it was predicted that c-Myc potentially interacted with the −2189/−2184 site of the *GOLM1* promoter and subsequently activated *GOLM1* transcription. MHCC-97H cells were cultured in conditions of 2% O_2_ for different durations, and chromatin immunoprecipitation (ChIP)-PCR analysis was performed. Results showed that c-Myc interacted with the *GOLM1* promoter, and the intensity of the DNA-protein interaction escalated significantly as the expression of c-Myc increased, which demonstrated that c-Myc positively modulated the transcription of *GOLM1* (Fig. 3a).

To verify the effect of c-Myc on the transcriptional activity of the *GOLM1* promoter, we constructed *GOLM1* promoter vector and site mutated mutant based on a luciferase reporter gene plasmid. This result indicated that overexpression of c-Myc significantly enhanced the transcriptional activity of *Luc2CP*, which lost its interaction with the *GOLM1* promoter when the binding site was mutated, which was consistent with the results of the ChIP analysis (Fig. 3b, S3a).

The above data demonstrated that the upregulation of c-Myc promoted the transactivation of GP73 in a mildly hypoxic microenvironment.

### Upregulation of GP73 promotes cell invasion

**Fig. 4:**
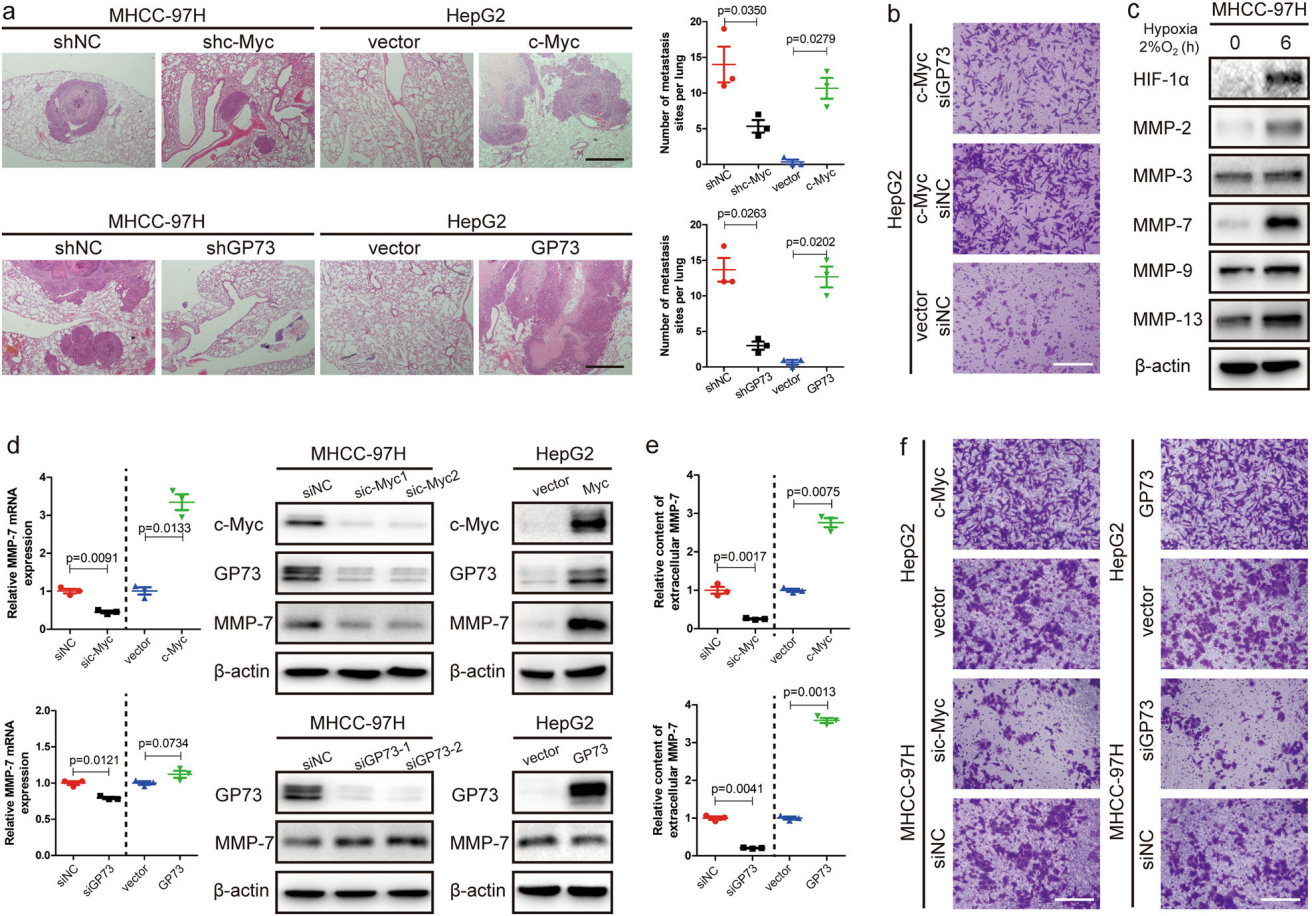
Upregulation of GP73 promotes cell invasion. **a** H&E staining of lung metastases in metastatic tumors from nude mice (scale bar: 1 mm). The lung metastases from the lungs of nude mice were counted and are shown as scatter diagrams. **b** HepG2 cells were transfected with siRNAs and vectors as described. Six hours after transfection, 100,000 cells from each group were subjected to Matrigel Transwell assays (scale bar: 100 μm). **c** MHCC-97H cells in the hypoxia group were cultured in a hypoxic chamber containing 2% oxygen, and cells in the control group were cultured in hypoxic chambers containing 21% oxygen. Each group was cultured for 6 h before the samples were harvested. The levels of the indicated proteins were determined by immunoblotting. **d** HepG2 cells were transfected with 2 μg pCMV-c-Myc, pCMV-GP73 or pCMV vector. MHCC-97H cells were transfected with a specific siRNA against c-Myc or GP73 or with a control siRNA. Samples were harvested 48 h after transfection. The mRNA level of MMP-7 was determined by qRT-PCR, and the levels of the indicated proteins were determined by immunoblot. **e** HepG2 cells were transfected with 2 μg pCMV-c-Myc, pCMV-GP73 or pCMV vector. MHCC-97H cells were transfected with c-Myc, GP73-specific siRNAs or control siRNAs. Cell culture supernatant was collected 48 h after transfection. The level of MMP-7 was determined by ELISA. **f** MHCC-97H and HepG2 cells were transfected with siRNAs and vectors as described. Six hours after transfection, 100,000 cells of each group were subjected to Matrigel Transwell assays (scale bar: 100 μm). Data in **a**, **d** and **e** are the mean ± s.e.m. and data represent three independent experiments. A two-tailed Student’s *t*-test was used for statistical analysis

Upregulation of c-Myc promoted GP73 transactivation and facilitated cancer metastasis under mildly hypoxic conditions; however, it was unknown whether c-Myc or GP73 directly induced cancer metastasis. Herein, an in vivo pulmonary metastasis model was established using HepG2 cells stably expressing c-Myc and MHCC-97H cells with stable knockdown of c-Myc compared with HepG2 cells stably expressing GP73 and MHCC-97H cells with stable knockdown of GP73. The results of the in vivo metastasis analysis revealed that both c-Myc and GP73 promoted cell metastasis. (Fig. 4a, S4a). To further determine whether c-Myc promote cell metastasis, we silenced GP73 in c-Myc-overexpressing HepG2 cells, and cell invasiveness was compared with that of c-Myc-overexpressing and negative control HepG2 cells (Fig. S4b). A Matrigel Transwell invasion assay demonstrated that cell invasiveness was sharply enhanced in c-Myc-overexpressing HepG2 cells, whereas invasiveness was slightly enhanced while GP73 was silenced in c-Myc-overexpressing HepG2 cells compared with negative control HepG2 cells. The abovementioned results indicated that both c-Myc and GP73 contributed to the process of cell invasion, but GP73 rather than c-MYC was the dominant cause of cell invasion (Fig. 4b, S4c). Most MMPs were upregulated under mildly hypoxic conditions, especially MMP-7 (Fig. 4c). Therefore, GP73 may accelerate cell invasion through regulation of MMP-7 expression under mildly hypoxic conditions.

We examined the level of MMP-7 mRNA while c-Myc and GP73 were mediated. We found that c-Myc could positively regulate the mRNA expression of MMP-7 and that GP73 could also positively regulate MMP-7 mRNA expression to some extent. Moreover, the expression of MMP-7 was detected by immunoblotting when c-Myc was mediated, which indicated that c-Myc also positively regulated the expression of MMP-7. We further mediated GP73 expression and surprisingly found that the protein level of intracellular MMP-7 was increased in GP73 knockdown cells and reduced in GP73-overexpressing cells, which was opposite to the change of mRNA level of MMP-7 (Fig. 4d). Since MMPs could be activated and released into extracellular spaces^23,24^, we measured supernatant MMP-7 level from cell culture media of c-Myc and GP73 overexpressing/knockdown cells by ELISA. Unlike the results derived from cell lysates, extracellular MMP-7 increased in c-Myc or GP73 overexpression cells and reduced in c-Myc or GP73 knockdown cells (Fig. 4e). Similar to the ELISA results, the Matrigel Transwell invasion assay demonstrated that cell invasion was enhanced in c-Myc- or GP73-overexpressing cells and weakened in c-Myc- or GP73-knockdown cells (Fig. 4f, S4d). Knockdown of GP73 induced an increase in intracellular MMP-7 but inhibited cell invasion, which suggested that inhibition of GP73 expression blocked the secretion of extracellular MMP-7 and GP73 might be involved in the trafficking of intracellular MMP-7.

### GP73 mediates the trafficking of intracellular MMP-7 and induces cell invasion

**Fig. 5:**
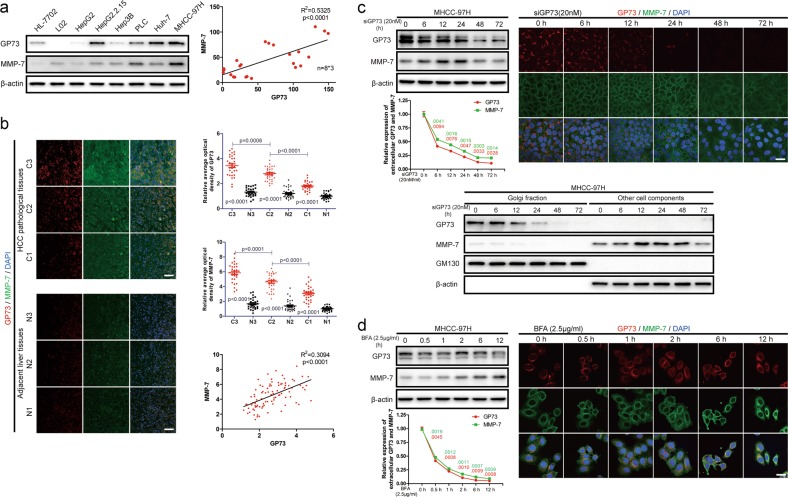
Inhibition of endogenous GP73 blocks trafficking of intracellular MMP-7. **a** The levels of GP73 and MMP-7 in indicated normal liver and HCC cell lines were determined by immunoblotting analysis. The correlation between levels of GP73 and MMP-7 in indicated cells was represented using linear correlation. **b** Immunofluorescence staining of GP73 (red) and MMP-7 (green) in primary tumor (*n* = 90) and adjacent liver (*n* = 90) tissues of HCC patients. Images are labeled by stage (1, 2, 3) and tissue type (C = primary tumor, *N* = adjacent liver, scale bar: 25 μm). The expression of specific proteins was measured using ImageJ and represented as the average optical density (AOD). The scatter plot indicates the relative expression of GP73 and MMP-7. The correlation between GP73 and MMP-7 in carcinoma tissues was represented using linear correlation. **c** Immunoblotting analysis and immunofluorescence staining (red: GP73, green: MMP-7; scale bar: 10 μm) of GP73 and MMP-7 after MHCC-97H cells were transfected with siGP73 (20 nM) for 0, 6, 12, 24, 48, and 72 h. Cell culture media were collected for ELISA before the cells were harvested. **d** Immunoblotting analysis and immunofluorescence staining (red: GP73, green: MMP-7; scale bar: 10 μm) of GP73 and MMP-7 after MHCC-97H cells were treated with BFA (2.5 μg/ml) for 0, 0, 5, 1, 2, 6, and 12 h. Cell culture media was collected for ELISA before cells were harvested. Data in **a**, **b**, **c** and **d** are the mean ± s.e.m. A two-tailed Student’s *t*-test was used for statistical analysis

To test the abovementioned hypothesis, the levels of GP73 and intracellular MMP-7 were determined in normal liver and HCC cell lines using immunoblotting analysis. It was manifested that GP73 correlated positively with intracellular MMP-7 (*R*^2^ = 0.5325), which implied GP73 potentially regulated the homeostasis of intracellular MMP-7 (Fig. 5a, S5a, b).

The levels and co-localization of GP73 and MMP-7 in primary tumor tissues and adjacent liver tissues (*n* = 90) were further examined using immunofluorescence staining. We demonstrated that both GP73 and MMP-7 were upregulated in primary tumor tissues, and, critically, MMP-7 was highly expressed in and near high GP73 expression cells, which suggested that GP73 was involved in the secretory process of MMP-7 from cytosol to extracellular spaces. Moreover, GP73 was correlated positively with MMP-7 in both tumor tissues and adjacent liver tissues (*R*^2^ = 0.3094) (Fig. 5b). The results above further convinced us that GP73 modulated trafficking of intracellular MMP-7 in tumor-related tissues.

The expression of endogenous GP73 was suppressed using GP73-specific siRNA for different durations, and immunoblotting and immunofluorescence analysis showed that MMP-7 accumulated in the cytosol throughout the 24 h following GP73-specific siRNA treatment of the cells. A longer time of treatment (beyond 24 h) with GP73-specific siRNA inhibited the intracellular MMP-7 accumulation, which suggested that a negative feedback loop might be triggered to eliminate excessive intracellular MMP-7. Extracellular MMP-7 was consistently reduced after GP73 silencing, which demonstrated that the trafficking of intracellular MMP-7 was inhibited as a consequence of GP73 inhibition. Interestingly, though the intracellular MMP-7 accumulated, Golgi apparatus-related MMP-7 levels was still reduced following GP73 silencing, which implied that GP73 silencing might inhibit the Golgi-localization of intracellular MMP-7. To further clarify the interaction of GP73 and intracellular MMP-7 during the process of vesicular trafficking, we cultured MHCC-97H cells in Dulbecco’s modified Eagle medium (DMEM) containing brefeldin A (BFA, 2.5 μg/ml). Immunoblotting and immunofluorescence analysis revealed that Golgi apparatus-dependent vesicular trafficking was inhibited after BFA stimulation and that MMP-7 accumulated in the cytosol, which supported that GP73 might play an essential role in vesicular trafficking of intracellular MMP-7. Twelve hours after BFA stimulation, GP73 localization overlapped with MMP-7 localization in the cytosol (Fig. 5d). The results above suggested that GP73 potentially interacted with MMP-7 through facilitating the vesicular trafficking of MMP-7.

**Fig. 6:**
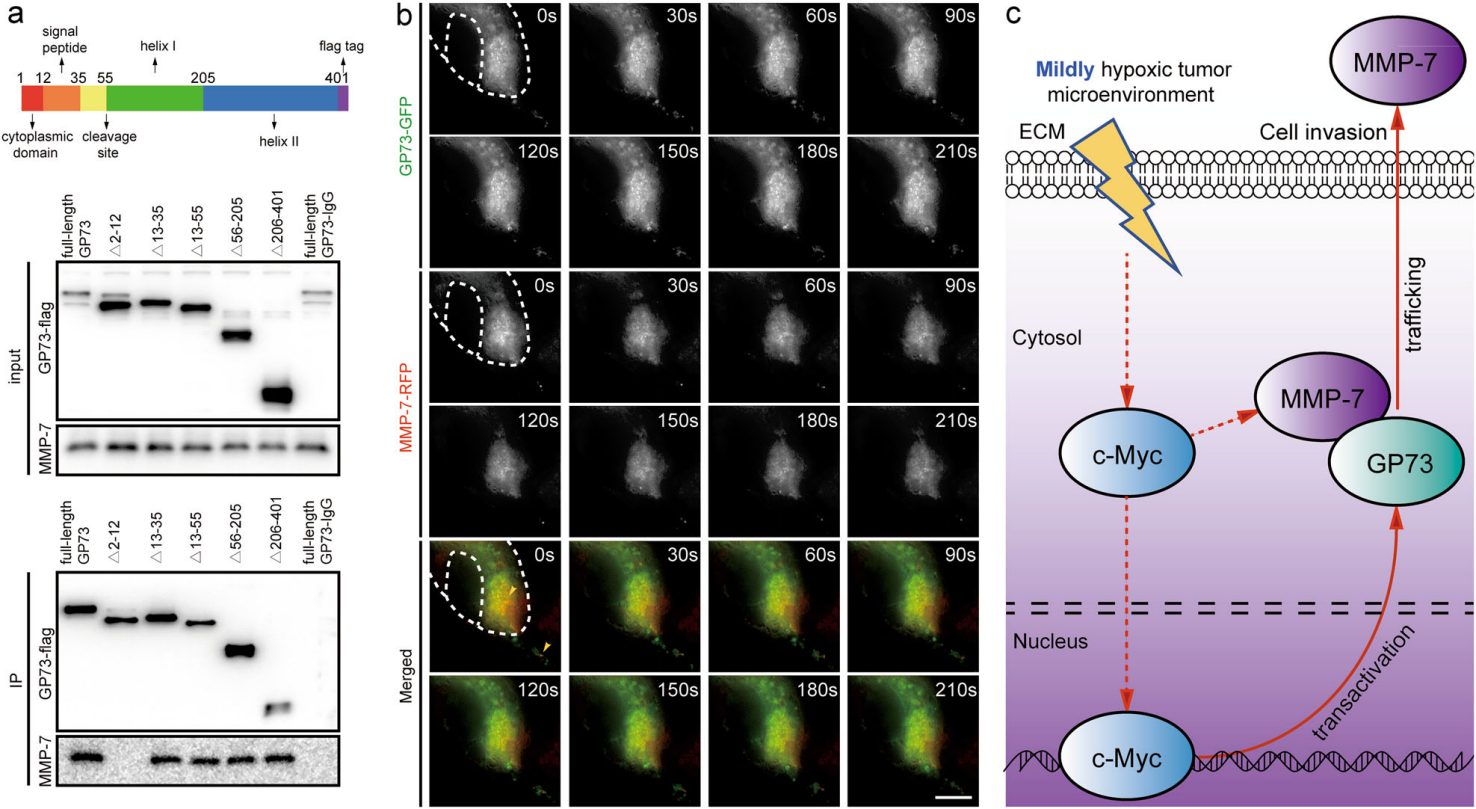
GP73 mediates the trafficking of intracellular MMP-7 and induces cell invasion. **a** 293 T cells were transfected with 2 μg pCMV-c-FLAG vector containing fragments of GP73 truncated mutants. Forty-eight hours after transfection, GP73 mutants and interacting proteins were co-immunoprecipitated with FLAG-tagged antibodies and blotted with the indicated antibodies. **b** 293 T cells were transfected with 1 μg pcDNA3.1-GP73-GFP and 1 μg pcDNA3.1-MMP-7-RFP vectors. Movements of GP73 (green) and MMP-7 (red) were captured every 5 s for 5 min (scale bar: 5 μm). **c** Overview of the GP73-mediated cell invasion process in a mildly hypoxic microenvironment: c-Myc transactivates GP73 and promotes metastasis of hepatocellular carcinoma cells through GP73-mediated MMP-7 trafficking in a mildly hypoxic microenvironment

Co-immunoprecipitation and immunoblotting analysis followed by GP73 mediation in HepG2 and MHCC-97H cells demonstrated that GP73 interacted with intracellular MMP-7 (Fig. S6a). To probe how they interacted on vesicular membranes, GP73 truncated mutants were constructed and expressed in 293 T cells. Co-immunoprecipitation and immunoblotting analysis demonstrated that GP73 lost its ability to interact with MMP-7 when amino acids 2–12 were deleted (Fig. 6a). The results above showed that GP73 interacted with MMP-7 in the region of its cytoplasmic domain, which demonstrated that GP73 is involved in the trafficking of MMP-7 through Golgi-associated vesicles. Moreover, we demonstrated that the expression of extracellular MMP-7 increased when full-length GP73 was overexpressed in 293 T cells, which suggested that only full-length GP73 is attributed to MMP-7 trafficking (Fig. S6b). In order to further test this hypothesis, GP73-GFP and MMP-7-RFP were expressed in 293 T cells, and live-cell imaging analysis was performed. GP73 co-localized with MMP-7 in the region of Golgi cisternae and intracellular vesicles, and the movement of GP73 and MMP-7 overlapped. MMP-7 was showed to separate from GP73 and released into extracellular spaces after their vesicular co-translocation from the cytosol to the plasma membrane (Fig. 6b).

The results collectively demonstrated that the expression of GP73 was activated in a mildly hypoxic microenvironment. GP73 is involved in vesicular trafficking of MMP-7, resulting in MMP-7 secretion and HCC cell invasion (Fig. 6c).

## Discussion

GP73, as a secretory protein highly expressed in HCC cells, has been regarded as an HCC-targeted diagnostic biomarker for decades. Recently, it has been reported that upregulation of GP73 promotes carcinogenesis and metastasis of HCC^17^. However, no study has indicated how GP73 transactivated in HCC cells. In our previous study, we cultured HepG2 cells under mildly hypoxic conditions and occasionally found that GP73 was upregulated. It is well known that the oxygen supply is insufficient in the tumor microenvironment, which obliges cancer cells to survive in a mildly hypoxic microenvironment^25,26^. Therefore, it has been posited that, GP73 is specifically upregulated in cancer cells in order to provide sufficient oxygen and promote metastasis in a mildly hypoxic microenvironment. We assessed the expression of hypoxia-related proteins and discovered that c-Myc was correlated positively with GP73 under hypoxic conditions with different oxygen concentrations. Then the expression of c-Myc was manipulated using a c-Myc expression vector and an shRNA vector targeting c-Myc. Results demonstrate that c-Myc positively regulates GP73 expression in vitro and in vivo. Immunochemical analysis further showed that c-Myc expression is abnormally upregulated and positively correlated with GP73 expression in pathological tissues derived from HCC patients. Since c-Myc is regarded as a common transcription factor, it appears that c-Myc might mediate the transactivation of GP73 in HCC cells. These analyses have demonstrated that c-Myc interacts with the promoter of *GOLM1* and subsequently activates its transcription.

It has been reported that GP73 promotes cell metastasis by modulating the expression of EMT-related proteins, and our study demonstrated that mildly hypoxic conditions activate the transcription of GP73^17,18^. Recent investigations have demonstrated that mildly hypoxic conditions stimulate the secretion of secretory proteins^27^. This evidence suggests that GP73 might promote the secretion of EMT-related secretory proteins, especially matrix metalloproteinases (MMPs). We measured the expression of MMPs under mildly hypoxic conditions, and the expression of MMP-7 was upregulated remarkably, indicating that MMP-7 might fulfill a dominant function in cell invasion under mildly hypoxic conditions. Moreover, we exhibited that the expression of MMP-7 is modulated by c-Myc or GP73 through c-Myc mediation and immunoblotting analysis. More importantly, c-Myc mediation exerted opposite modulatory effects on intracellular MMP-7 expression compared with that of GP73 mediation, which suggests that c-Myc participates in the regulation of MMP-7 expression and that GP73 might be involved in the trafficking of intracellular MMP-7. Trafficking-related assays and co-immunoprecipitation assays have demonstrated that GP73 interacts with MMP-7 in the region of the cytoplasmic domain and assists in trafficking of intracellular MMP-7. Inhibition of GP73 blocks the trafficking of intracellular MMP-7 and leads to repression of cancer cell invasion. Furthermore, in our recent study, it has been proved that GP73 is involved in the trafficking of intracellular MMP-2, a factor playing comparable functional roles to MMP-7^19^. Both of the two factors interact with GP73 in the region of cytoplasmic domain, therefore MMPs might act as potential substrates for GP73-mediated protein trafficking and facilitate HCC metastasis.

This study has provided two potential targets to combat HCC. On the one hand, it would not be advantageous to manipulate the expression of c-Myc because it plays pivotal roles in cell cycle regulation, cell differentiation and apoptosis in normal cells. On the other hand, GP73 deletion has little impact on cell cycle and metabolism of normal cells as has been shown with GP73 knockout mice^17^. Therefore, it is supposed that knockdown of GP73 using specific siRNAs might provide a relatively safe approach to inhibit HCC metastasis. Moreover, GP73-targeted siRNAs are expected to be potential therapeutic drugs targeting metastatic HCC.

In summary, this study has indicated how GP73 is transactivated in HCC cells and has further shown that GP73 plays functional roles in the trafficking of EMT-related proteins. We have discovered a mechanism that strongly appears to assist the survival and metabolism of cancer cells in a mildly hypoxic tumor microenvironment, and it might provide a potential target for combating metastatic HCC.

## Materials and methods

### Cell culture and inducing a mildly hypoxic condition

MHCC-97H cells were from the Liver Cancer Institute (Zhongshan Hospital, Fudan University, China), HepG2 and 293 T cells were from the American Type Culture Collection (ATCC, Manassas, VA, USA). Cells were cultured in DMEM (Thermo Fisher, Carlsbad, CA, USA) supplemented with 10% foetal bovine serum (FBS, Thermo Fisher) in 5% CO_2_ at 37 °C. Mild hypoxia was induced by placing HepG2 and MHCC-97H cells in a hypoxic chamber (Mitsubishi Gas Chemical Company, Inc, Japan) containing 0–21% O_2_ at 37 °C. Cell lines above were authenticated by STR profiling at Cobioer Bioscience Co., Ltd. (Nanjing, China) and experiments were performed within < 10 passages after authentication.

### Immunoblotting

Cells were lysed using RIPA lysis buffer (Millipore, Billerica, MA, USA) with a protease and phosphatase inhibitor cocktail (Thermo Fisher). Samples were subjected to immunoblotting as previously described^28^. The transfer process was performed using a cellulose nitrate membrane (0.22 μm, Millipore) under constant current conditions of 200 mA for 2 h. Antibodies against GP73 were purchased from Thermo Fisher (1:3000); antibodies against c-Myc, HIF-1α, MMP-2, MMP-3, MMP-7, MMP-9, MMP-13 and GM130 were purchased from Cell Signaling Technology, Danvers, MA, USA (1:2000); antibodies against FLAG-tag and β-actin were purchased from EarthOX, San Francisco, CA, USA (1:5000).

### RNA isolation and quantitative real-time PCR

RNA was isolated from cells using TRIzol reagent (Thermo Fisher) following the manufacturer’s instructions. cDNA was generated using the PrimeScript™ RT Reagent Kit with gDNA Eraser (TaKaRa, Dalian, China) according to the manufacturer’s instructions. mRNA expression was quantified via qRT-PCR using a 7500 Real-Time PCR system (Applied Biosystems, Foster City, CA, USA). qRT-PCR was performed using SYBR Premix Ex Taq® (TaKaRa) with the primers listed in Supplementary Table 1a. The parameters of qRT-PCR were as specified in the manufacturer’s instructions. The mRNA expression of β-actin was used as a reference.

### Plasmid and siRNA transfection

The plasmids pCMV-c-Myc and pCMV-GP73 were purchased from Sinobiological (Beijing, China). Plasmids (1 μg) were transfected into cells using Lipofectamine 3000 reagent (Thermo Fisher). Cells were resuspended for subsequent experiments 48 h after transfection. The siRNAs corresponding to human c-Myc (MYC) and GP73 (GOLM1) were purchased from GenePharma (Shanghai, China). Details on the siRNAs are shown in Supplementary Table 1b. SiRNAs were transfected into cells using Lipofectamine RNAiMAX reagent (Thermo Fisher) at a final concentration of 20 nM. Cells were resuspended for subsequent experiments 48 h after transfection.

### Xenograft model

HepG2 cells stably expressing c-Myc were constructed using the pCMV-blank plasmid (Beyotime, Nanjing, China). MHCC-97H cells with stable knockdown of c-Myc were constructed and screened using the pLKO.1-turbo-GFP plasmid (details are shown in Supplementary Table 1c). HepG2 cells were transfected with pCMV-blank or pCMV-c-Myc vectors and screened using hygromycin (100 μg/ml) for 10 days, and MHCC-97H cells were transfected with pLKO.1-turbo-GFP or pLKO.1-turbo-GFP-shc-Myc vectors and screened using puromycin (2 μg/ml) for 7 days. Positive colonies were identified using qRT-PCR and immunoblotting analysis. Stably transfected HepG2 cells were maintained using DMEM containing hygromycin (30 μg/ml) and stably transfected MHCC-97H cells were maintained using DMEM containing puromycin (1 μg/ml). Nude mouse (4 weeks old, male, Slac Laboratories, Shanghai, China) were randomized and xenograft models generated by subcutaneously injecting 5 × 10^6^ cells (MHCC-97H^shV^ and MHCC-97H^shc-Myc^) or 7 × 10^6^ cells (HepG2^vector^ and HepG2^c-Myc^). Animals were ramdonsacrificed 3 weeks after injection of tumor cells, and tumors were excised for immunohistochemical analysis to detect c-Myc and GP73. All procedures for animal care and use were in compliance with the Guide for the Care and Use of Laboratory Animals (NIH, 8^th^ edition).

### In vivo metastasis assay

HepG2 cells stably expressing GP73 were constructed using the pCMV-blank plasmid. MHCC-97H cells with stable knockdown of GP73 were constructed based on the pLKO.1-turbo-GFP plasmid (details are shown in Supplementary Table 1c). HepG2 cells were transfected with pCMV-blank or pCMV-GP73 vectors and screened using hygromycin (100 μg/ml) for 10 days, and MHCC-97H cells were transfected with pLKO.1-turbo-GFP or pLKO.1-turbo-GFP-shGP73 vectors and screened using puromycin (2 μg/ml) for 7 days. Positive colonies were identified using qRT-PCR and immunoblotting analysis. Stably transfected HepG2 cells were maintained using DMEM containing hygromycin (30 μg/ml) and stably transfected MHCC-97H cells were maintained using DMEM containing puromycin (1 μg/ml). NOD-SCID mouse (4 weeks old, male, Slac Laboratories) were randomized and in vivo metastasis models were generated by injecting 2 × 10^6^ cells (GP73 or c-Myc stably knockdown/expressed cells) via the tail vein. Animals were sacrificed 70 days after tumor cell injection, and their lungs were excised for imaging and haematoxylin and eosin (H&E) staining. All procedures for animal care and use were in compliance with the Guide for the Care and Use of Laboratory Animals (NIH, 8^th^ edition).

### Immunochemical analysis and immunofluorescence staining of HCC tissues derived from patients

Carcinoma and adjacent liver tissues (*n* = 90) derived from HCC patients were from the First Affiliated Hospital, Zhejiang University School of Medicine (Hangzhou, China). The Institutional Review Board at Zhejiang University School of Medicine approved the protocol of this study, and all patients provided informed consent. Samples were fixed in 4% formalin and embedded in paraffin. Sections with a thickness of 4 μm were placed on glass slides for immunochemical or immunofluorescence staining analysis. Immunochemical or immunofluorescence staining analysis was performed as previously reported^28^. Endogenous GP73 was tagged using mouse anti-human GP73 antibody (Abvona, Taipei, Taiwan), c-Myc was tagged using rabbit anti-human c-Myc antibody, Ki-67 was tagged using rabbit anti-human Ki-67 antibody and MMP-7 was tagged using rabbit anti-human MMP-7 antibody (Cell Signaling Technology). Images were captured using an Olympus FV1000 confocal microscope (Olympus Corporation, Japan).

### Luciferase reporter analysis

The fragments of promoter of *GOLM1* and the fragments of binding site mutated mutant were synthesized by Oligobio (Beijing, China) and inserted into pGL4.19. HepG2 cells were co-transfected with pGL4.19 containing promoter of *GOLM1*/binding site mutated mutant, pRL-TK and vector/c-Myc. Forty-eight hours after transfection, cells were harvested and lysed using passive lysis buffer. Luciferase activity was analyzed using a Dual Luciferase Reporter Assay System kit (Promega, Madison, WI, USA) according to the manufacturer’s instructions. Total light intensity was measured with a SpectraMax M5 microplate reader (Molecular Devices, Sunnyvale, CA, USA).

### Chromatin immunoprecipitation and PCR analysis

The binding site of c-Myc to the promoter of GOLM1 was predicted using the JASPAR database (http://jaspar.genereg.net). ChIP analysis was performed using a SimpleChIP Enzymatic Chromatin IP kit (Cat#9003, Cell Signaling Technology) following the manufacturer’s instructions. DNA–protein complexes were immunoprecipitated using a specific antibody against c-Myc (Cell Signaling Technology). Immunoprecipitated DNA fragments and input DNA were used as templates for PCR with PrimeSTAR GXL polymerase (TaKaRa). Details on the primers are shown in Supplementary Table 1d.

### Matrigel Transwell invasion assay

Matrigel (Corning, NY, USA) was mixed with 1 × PBS at 1:5. Transwell chambers were covered using 50 μL of the mixture. The plates were allowed to equilibrate in 5% CO_2_ at 37 °C for 4 h. Cells were harvested 48 h after transfection with c-Myc/GP73 plasmids or specific siRNAs. Every chamber was seeded with 100,000 cells diluted in 100 μL serum-free DMEM. The bottom of the well was filled with 800 μL DMEM with 10% FBS. Cells were incubated in 5% CO_2_ at 37 °C for an additional 24 h and fixed with methanol (>99.5%) for 5 m. After staining was conducted with 0.3% crystal violet, cells in the upper chamber were removed. Images were captured by bright-field microscopy using an Olympus DP70 microscope (Olympus Corporation).

### Co-immunoprecipitation assay

In total 293 T cells in 6-well plates were transfected with pCMV and pCMV-GP73 vectors. After 48 h of transfection, cells were harvested, and lysed on ice for 30 min using 500 μL of 1 × RIPA lysis buffer, and centrifuged at 15,000 × *g* for 15 min. The supernatants were collected and split into 2 equal aliquots. Then, 30 μL magnetic beads (Thermo Fisher) and 5 μL antibodies (anti-GP73 mAb and normal rabbit IgG) were added. The mixture was incubated at 4 °C overnight. Purified proteins were washed three times with 1 × RIPA lysis buffer and eluted using IP elution buffer (5 mM glycine, pH = 2.7). Samples were denatured by adding 5 × loading buffer and boiling for 5 min. Interaction of GP73 and MMP-7 was detected using an immunoblotting assay.

### Mapping of the binding site of GP73/MMP-7 in vitro

Truncated mutants of GP73 were designed, as shown in Fig. 6a. Mutants were constructed based on the pCMV-c-FLAG plasmid. Protein extraction, immunoprecipitation and immunoblotting were performed, as described above. Truncated mutants of GP73 were immunoprecipitated using rabbit anti-FLAG pAb and detected using mouse anti-FLAG mAb (Sigma-Aldrich Co., St. Louis, MO, USA).

### Live-cell imaging

We constructed pcDNA3.1-GP73-GFP and pcDNA3.1-MMP-7-RFP vectors based on pcDNA3.1-GFP and pcDNA3.1-RFP vectors. 293 T cells in 3 cm confocal microscopy dishes were co-transfected with 1 μg pcDNA3.1-GP73-GFP vector and 1 μg pcDNA3.1-MMP-7-RFP vector. After 48 h of transfection, fusion proteins were tracked using a Structural Illumination Microscope (SIM, Nikon Corporation, Japan). Images were captured every 5 s for 5 m.

### Statistical analysis

The values were analyzed using a two-tailed Student’s *t*-test and presented as the mean ± standard error of the mean (SEM). Statistical analysis was performed using the software Statistical Package for the Social Sciences (SPSS) version 16.0. *P*-values < 0.05 were considered significant.

## Supplementary information

Supplementary Materials and Methods

Supplementary Tables

Supplementary Figures
