# Supplementary material for: c-Myc transactivates GP73 and promotes metastasis of hepatocellular carcinoma cells through GP73-mediated MMP-7 trafficking in a mildly hypoxic microenvironment
Source: Oncogenesis. 2019 Oct 7;8(10):58. doi: 10.1038/s41389-019-0166-7 (PMC6779757; doi:10.1038/s41389-019-0166-7)
Supplement: Supplementary file 1 — Supplementary Materials and Methods [file 41389_2019_166_MOESM1_ESM.docx]

**Supplementary Information**

**Supplementary Materials and Methods**

**Wound healing assay**

The cell culture media were replaced with serum-free DMEM while the cells were at 100% confluence. The cells were incubated in 5% CO_2_ at 37˚C for an additional 12 h. The cells were wounded using a 10 μL micropipette tip and washed using 1×PBS. Next, the cells were supplemented with serum-free DMEM and incubated in 5% CO_2_ at 37˚C. Images were captured by bright-field microscopy using an Olympus DP70 microscope (Olympus Corporation, Japan).

**Extracellular GP73 and MMP-7 determination analysis**

The cell culture media were replaced with serum-free DMEM 36 h after cells were transfected with c-Myc/GP73 plasmids or specific siRNAs and cultured for an additional 24 h. The cell culture media were then collected; extracellular GP73 was measured using a human GOLM1/GP-73 ELISA kit (Raybiotech, Norcross, GA, USA), and extracellular MMP-7 was determined using a human MMP-7 ELISA kit (Raybiotech), following the manufacturer’s instructions in both cases.

**Isolation of Golgi fraction**

MHCC-97H cells in 15 cm cell culture dishes (1×10^7^ cells per dish) were collected after transfected with siGP73 (20 nM) for 0, 6, 12, 24, 48, and 72 h. Cells were washed with ice-cold PBS and 10 mL of 0.25 M sucrose solution, then suspended in 300 μL of 0.25 M sucrose solution. Cells were ground using a homogenizer and Golgi fraction was isolated from other cell components using a Golgi Isolation Kit (Sigma-Aldrich Co., St. Louis, MO, USA) following the manufacturer’s instructions. Proteins in Golgi fraction and other cell components were examined using immunoblotting analysis.
