# Supplementary material for: c-Myc transactivates GP73 and promotes metastasis of hepatocellular carcinoma cells through GP73-mediated MMP-7 trafficking in a mildly hypoxic microenvironment
Source: Oncogenesis. 2019 Oct 7;8(10):58. doi: 10.1038/s41389-019-0166-7 (PMC6779757; doi:10.1038/s41389-019-0166-7)
Supplement: Supplementary file 2 — Supplementary Tables [file 41389_2019_166_MOESM2_ESM.docx]

**Supplementary Information**

**Supplementary Tables**

**Supplementary Table 1**

1. **Primer sequences for quantitative realtime-PCR**

| Primers | Sequences (5’-3’) |
| --- | --- |
| GP73 | F: CAGCGCTGATTTTGAGATGAC |
|  | R: ATGATCCGTGTCTGGAGGTC |
| c-Myc | F: GGCTCCTGGCAAAAGGTCA |
|  | R: CTGCGTAGTTGTGCTGATGT |
| MMP-7 | F: ACATCATGATTGGCTTTGCGCGAG |
|  | R: TCCCATACCCAAAGAATGGCCAAG |
| Luciferase | F: TACGTTAACAACCCCGAGGC |
|  | R: TCCACGATCTCCTTCTCGGT |
| β-actin | F: TTCCAGCCTTCCTTCCTGGG |
|  | R: TTGCGCTCAGGAGGAGCAAT |

1. **Sequences of siRNAs**

| Targets | Sense and antisense chains (5’-3’) |
| --- | --- |
| siGP73#1 | S: GAACAGUGUGAGGAGCGAATT |
|  | A: UUCGCUCCUCACACUGUUCTT |
| siGP73#2 | S: GUUGAGAAAGAGGAAACCATT |
|  | A: UGGUUUCCUCUUUCUCAACTT |
| sic-Myc#1 | S: CCUGAGACAGAUCAGCAACTT |
|  | A: GUUGCUGAUCUGUCUCAGGTT |
| sic-Myc#2 | S: CCUGAGACAGAUCAGCAACTT |
|  | A: GUUGCUGAUCUGUCUCAGGTT |
| siNC | S: GCGACGAUCUGCCUAAGAUTT |
|  | A: AUCUUAGGCAGAUCGUCGCTT |

*Sequences of siRNAs were based on shRNA sequences targeting specific target shown on the website of Sigma-Alderich.

1. **Primer sequences of shRNAs for construction of stably-transfected cell lines**

| Primers | Sequences (5’-3’) |
| --- | --- |
| shGP73#1 | **F:GAATTC**CCGGGAACAGTGTGAGGAGCGAATACTCGAGTATTCGCTCCTCACACTGTTCTTTTTTGACCGGT |
| shGP73#2 | **F:GAATTC**CCGGGTTGAGAAAGAGGAAACCAATCTCGAGATTGGTTTCCTCTTTCTCAACTTTTTTGACCGGT |
| shc-Myc#1 | **F:GAATTC**CCGGCCTGAGACAGATCAGCAACAACTCGAGTTGTTGCTGATCTGTCTCAGGTTTTTGACCGGT |
| shc-Myc#2 | **F:GAATTC**CCGGCCTGAGACAGATCAGCAACAACTCGAGTTGTTGCTGATCTGTCTCAGGTTTTTGACCGGT |
| shV | Empty pLKO.1-turbo-GFP vector |

*Sequences of shRNAs were from the website of Sigma-Alderich. In the process of stably-knockdown cell lines construction, it was found the knockdown efficiencies of shGP73#2 and shc-Myc#2 were low. Therefore, only shGP73#1 and shc-Myc#1 were applied in *in vivo* analysis. The sequences were the same to siRNAs shown in Supplementary Table 1A and off-target effect was examined using immunoblotting in *in vitro* analysis.

1. **Primers for ChIP analysis**

| Primers | Sequences (5’-3’) |
| --- | --- |
| *GOLM1* promoter (-2189/-2184) | F: CATAGAAGCAGAATGCTAT |
|  | R: AACACTGTTGCACTGGAGA |
