# Supplementary material for: c-Myc transactivates GP73 and promotes metastasis of hepatocellular carcinoma cells through GP73-mediated MMP-7 trafficking in a mildly hypoxic microenvironment
Source: Oncogenesis. 2019 Oct 7;8(10):58. doi: 10.1038/s41389-019-0166-7 (PMC6779757; doi:10.1038/s41389-019-0166-7)
Supplement: Supplementary file 3 — Supplementary Figures [file 41389_2019_166_MOESM3_ESM.docx]

**Supplementary Information**

**Supplementary Figures**

**
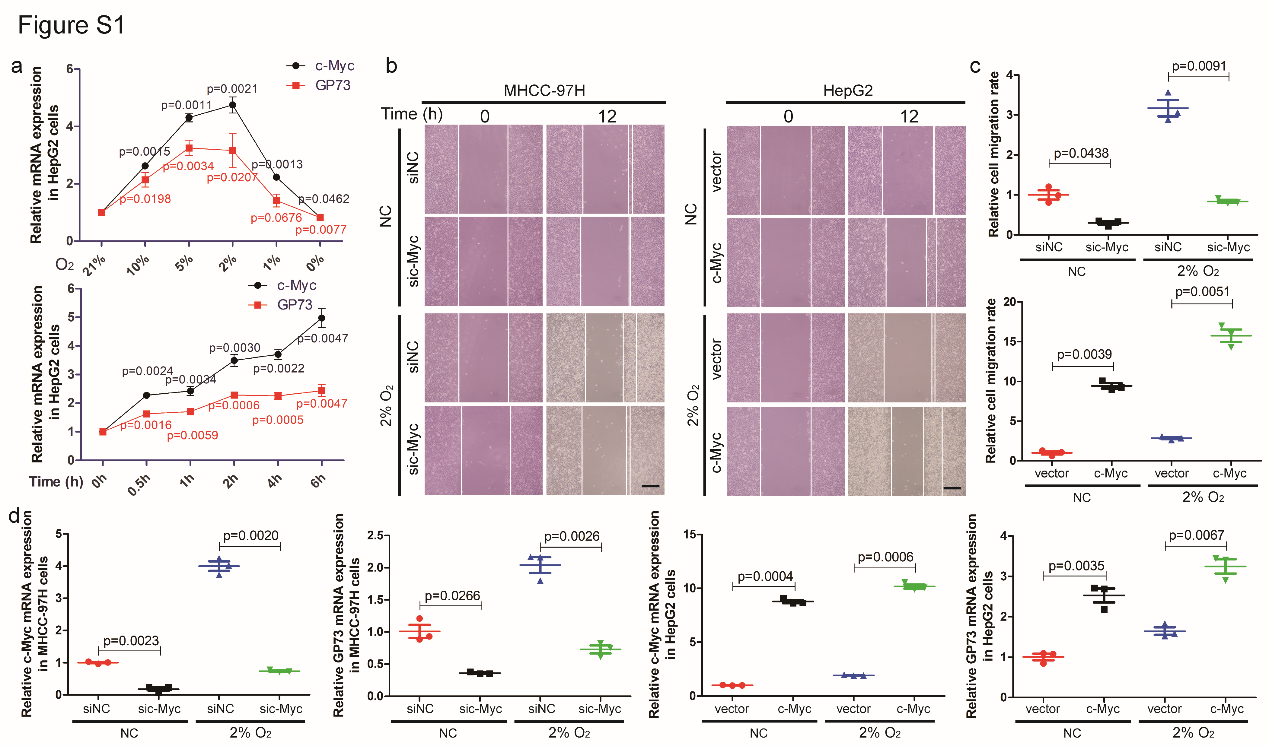
**

**Figure S1. Upregulation of c-Myc and GP73 promotes cell metastasis in a mildly hypoxic microenvironment.**

1. HepG2 cells were incubated in a hypoxic chamber containing 0%, 1%, 2%, 5%, 10% or 21% oxygen for 6 h before samples were harvested. Then, the HepG2 cells were incubated in a hypoxic chamber containing 2% oxygen for 0, 0.5, 1, 2, 4 or 6 h before samples were harvested. The level of indicated mRNA expression was determined by quantitative real-time PCR (qRT-PCR).
2. HepG2 cells were transfected with 2 μg pCMV-c-Myc or pCMV vector. MHCC-97H cells were transfected with GP73-specific siRNAs or control siRNAs. Forty-eight hours after transfection, cells were cultured in serum-free culture media and treated with 2% or 21% oxygen for an additional 12 h, and images were captured (scale bar: 100 μm).
3. The migration of MHCC-97H cells in **b** was measured and expressed as the relative cell migration rate.
4. HepG2 cells were transfected with 2 μg pCMV-c-Myc or pCMV vector. MHCC-97H cells were transfected with c-Myc-specific siRNAs or control siRNAs. Forty-eight hours after transfection, the cells were treated with 2% or 21% oxygen for an additional 12 h before samples were harvested. The levels of the indicated mRNA expression were determined by qRT-PCR. Data in **a, c** and **d** are the mean±s.e.m. and represent three independent experiments. A two-tailed Student’s *t*-test was used for statistical analysis.

**
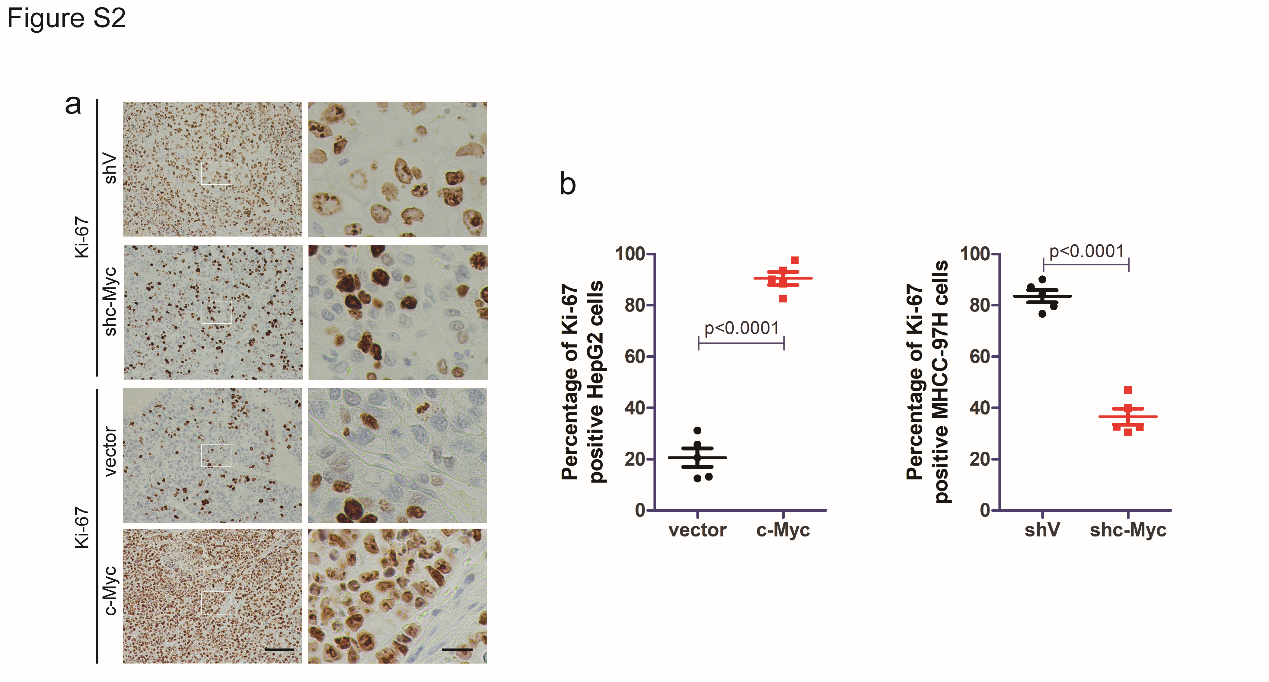
**

**Figure S2. c-Myc positively modulates GP73 expression and promotes tumor progression *in vivo*.**

1. Immunohistochemical analysis of Ki-67 in tumors derived from nude mice bearing xenografts (scale bar: left 50 μm, right 10 μm).
2. The scatter plot indicates the relative expression of Ki-67. Data in **b** are the mean±s.e.m. A two-tailed Student’s *t*-test was used for statistical analysis.

**
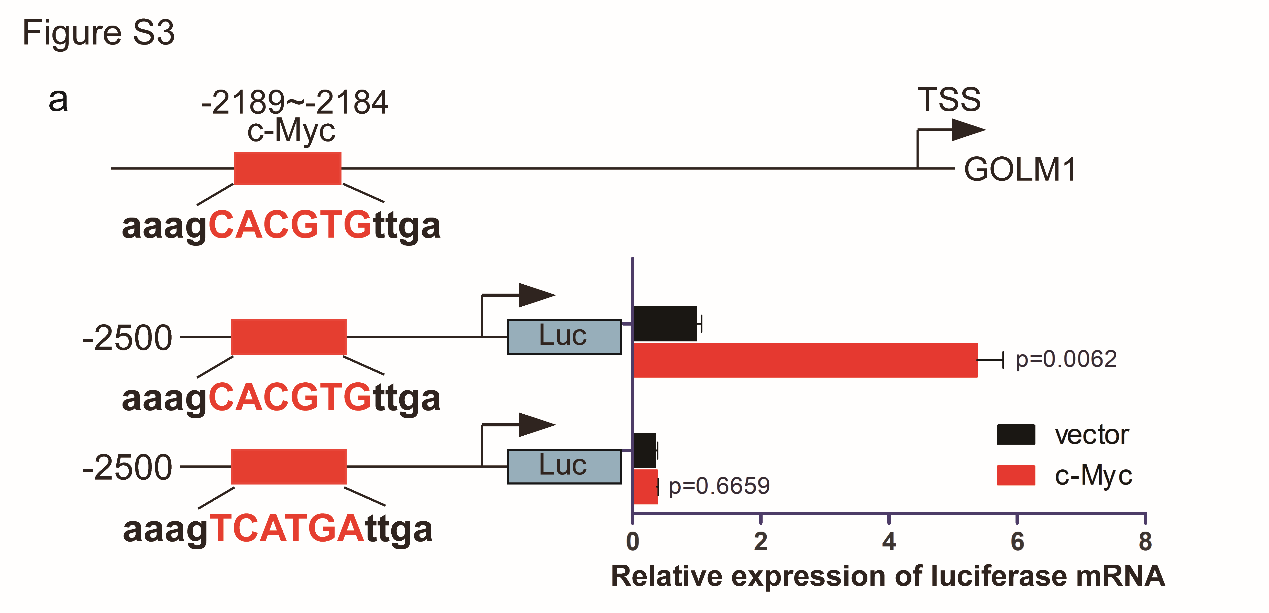
**

**Figure S3. Upregulation of c-Myc promotes transactivation of GP73.**

1. Luciferase mRNA expression was measured using qRT-PCR after HepG2 cells were transfected with pGL4.19 containing promoter of *GOLM1*/binding site mutated mutant, pRL-TK and vector/c-Myc. Data in **a** are the mean±s.e.m., and data represent three independent experiments. A two-tailed Student’s *t*-test was used for statistical analysis.

**
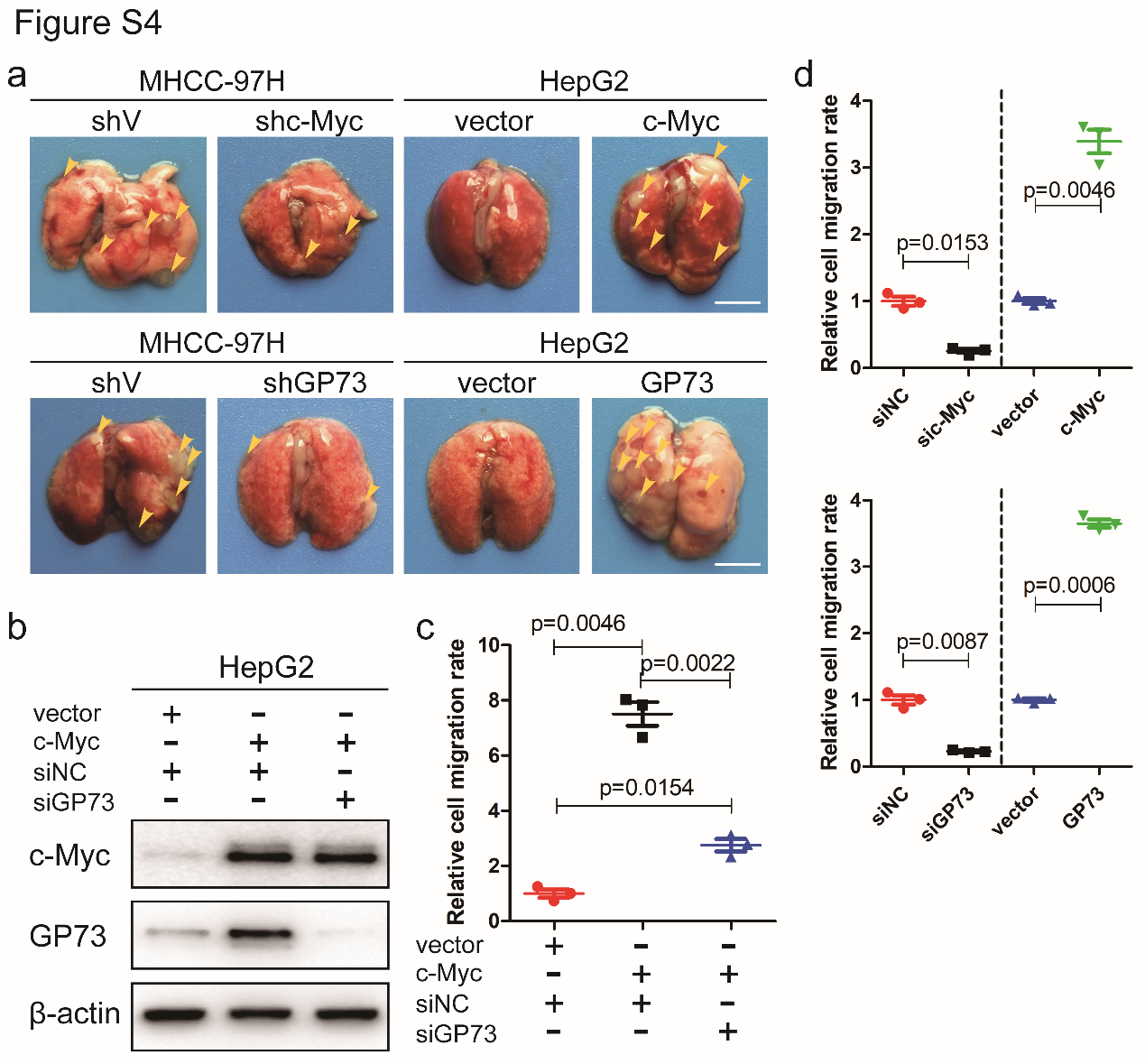
**

**Figure S4. Upregulation of GP73 promotes cell invasion.**

1. Images of lung metastases of metastatic tumors derived from nude mice intravenously injected with HepG2^vector^, HepG2^c-Myc^, MHCC-97H^shV^, MHCC-97H^shc-Myc^, HepG2^GP73^, and MHCC-97H^shGP73^ cells (scale bar: 5 mm).
2. HepG2 cells were transfected with vectors and siRNAs as described. Samples were harvested 48 h after transfection and the levels of the indicated proteins were determined by immunoblot.
3. The scatter plot shows the relative cell migration rate of cells in Fig. 4b.
4. The scatter plot shows the relative cell migration rate of cells in Fig. 4f. Data in **c** and **d** are the mean±s.e.m. and represent three independent experiments. A two-tailed Student’s *t*-test was used for statistical analysis.

**
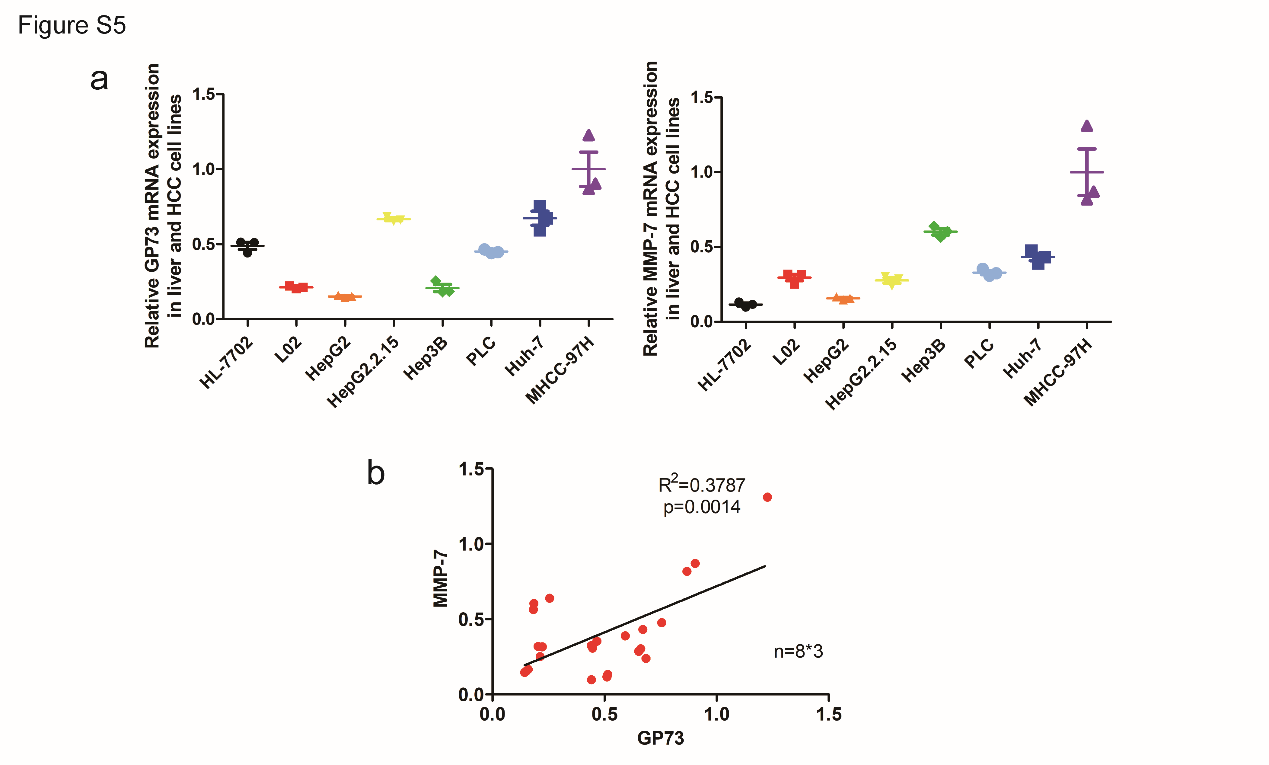
**

**Figure S5. GP73 correlates positively with MMP-7 in HCC cell lines.**

1. The mRNA levels of GP73 and MMP-7 in indicated normal liver and HCC cell lines were determined by qRT-PCR.
2. The correlation between mRNA levels of GP73 and MMP-7 in indicated cells was represented using linear correlation. Data in **a** and **b** are the mean±s.e.m. A two-tailed Student’s *t*-test was used for statistical analysis.

**
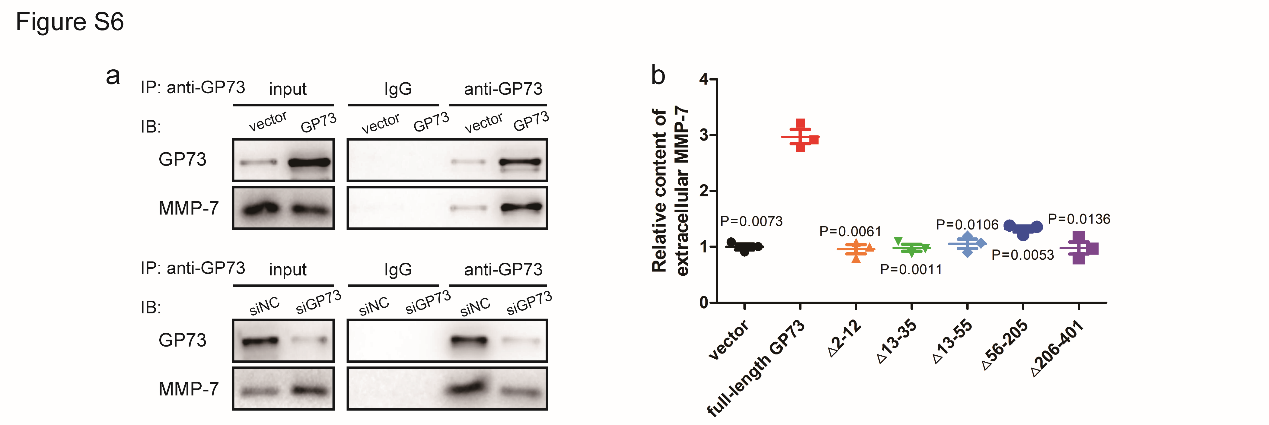
**

**Figure S6. GP73 mediates the trafficking of intracellular MMP-7 and induces cell invasion.**

1. HepG2 cells were transfected with 2 μg pCMV-GP73 or pCMV vector. MHCC-97H cells were transfected with c-Myc-specific siRNAs or control siRNAs. Forty-eight hours after transfection, GP73 and interacting proteins were co-immunoprecipitated with GP73 antibody and blotted with the indicated antibodies.
2. 293T cells were transfected with 2 μg pCMV-c-FLAG vector containing fragments of GP73 truncated mutants. Forty-eight hours after transfection, cell culture media were collected, and the expression of extracellular MMP-7 was determined using an ELISA kit targeting MMP-7. Data in **b** are the mean±s.e.m., and data represent three independent experiments. A two-tailed Student’s *t*-test was used for statistical analysis.
